# Responsiveness to inhibitory signals changes as a function of colony size in honeybees (*Apis mellifera*)

**DOI:** 10.1098/rsif.2021.0570

**Published:** 2021-11-10

**Authors:** Heather C. Bell, Kevin Hsiung, Patrick Pasberg, Frédéric D. Broccard, James C. Nieh

**Affiliations:** ^1^ Division of Biological Sciences, Section of Ecology, Behavior, and Evolution and, University of California San Diego, 9500 Gilman Drive, La Jolla, CA 92093, USA; ^2^ Institute for Neural Computation, University of California San Diego, 9500 Gilman Drive, La Jolla, CA 92093, USA; ^3^ Department of Mechanical Engineering, Section of Biomimetics, Westphalian University of Applied Sciences, Münsterstrasse 265, 46397 Bocholt, Germany

**Keywords:** honeybee, communication signals, superorganism, stop signal, colony size, adaptation

## Abstract

Biological collectives, like honeybee colonies, can make intelligent decisions and robustly adapt to changing conditions via intricate systems of excitatory and inhibitory signals. In this study, we explore the role of behavioural plasticity and its relationship to network size by manipulating honeybee colony exposure to an artificial inhibitory signal. As predicted, inhibition was strongest in large colonies and weakest in small colonies. This is ecologically relevant for honeybees, for which reduced inhibitory effects may increase robustness in small colonies that must maintain a minimum level of foraging and food stores. We discuss evidence for size-dependent plasticity in other types of biological networks.

## 1. Introduction

Researchers have long noted that colonies of eusocial organisms, like honeybees, behave collectively as part of a superorganism (the colony) similarly to cells within a multicellular organism [1–4]. As in many other biological systems, members of honeybee colonies make contact with only a few others in a given span of time, are distributed across space and are heterogeneous with respect to a number of parameters. Each bee makes decisions based on locally available information in accordance with its unique responsiveness to that information, which is a function of both internal factors, such as genetics and motivational state, and external factors, like the quality of a particular food source. The outcome of decisions is shared via excitatory or inhibitory signals with a few close neighbours [4–8].

Honeybees use the excitatory *waggle dance* to recruit hivemates to favourable resources, such as food sites [9]. However, if a forager is attacked at a food source or experiences deteriorating conditions, it returns to the nest and produces weakly inhibitory *stop signals* directed at dancers advertizing that location [10–12]. Stop signals elicit a brief pause from waggle dancers and reduce the probability that a waggle dancer will continue waggle dancing. Individual stop signals have a low associated probability of completely halting a waggle dance. However, they cumulatively inhibit recruitment [12,13]. The interplay between waggle dances and stop signals allows honeybee colonies to make collective decisions that have intriguing emergent properties [14–16]. For example, when a potential nest site is being advertized, the colony must rapidly coalesce around the correct decision. Dancers for different nest sites compete, with the longest lasting dance performances winning out. However, between dances, the dancers also perform stop signals that target dancers for different nest sites. The resulting cross-inhibition shortens the dancing process, allows the colony to more rapidly choose the best site, and increases the reliability of this system by overcoming deadocks—all without any central director [15].

Honeybee colonies respond to changing conditions, such as food availability outside and inside the nest. Foraging patterns shift throughout the day and across seasons in response to changes in floral availability and the risk of foraging on these resources. We propose that honeybee colonies can modify their reactions to unfavourable conditions in the environment, depending on multiple factors. We postulate that the mechanism for this threshold is experience-dependent plasticity in the sensitivity of individuals to stop signals. Specifically, we predict that the more stop signals received by an individual, the less responsive it becomes to future signals as a result of behavioural habituation [17]. The logic underlying this prediction, at the colony level, is that it would not be beneficial for colonies to curtail recruitment to all sites at times when every site is unfavourable to some degree (such as during times of high predator presence at most food resources). At these times, colonies should have a high level of stop signalling.

Other arthropods habituate to vibrations [18,19]; although to our knowledge, habituation to an intraspecific vibrational communication signal has not been investigated. Honeybees habituate to other biologically relevant stimuli, such as antennal stimulation with sucrose [20], and bumblebees habituate to novel visual stimuli after repeated exposure during foraging [21]. Our preliminary observations suggested that honeybees can become unresponsive to stop signals, perhaps as a result of habituation (see electronic supplementary material).

Due to the significant limitation that stop signals need to be identified using both behavioural and acoustic characteristics [22], it is difficult to estimate, in a typical colony with thousands of individuals, if and how stop signalling levels change over time. Colony-wide automated vibration detection is a useful tool, but noise and the conflating factors of multiple signals sharing similar spectral properties can limit such detection [22,23]. However, Smith & Chen [24] demonstrated that the overall level of detectable vibrational signalling is inversely related to colony size. We analysed data from a separate colony survey study, and found that colony size and stop signalling were inversely related when we controlled for the level of activity of the colony (see Material and methods). Therefore, manipulating colony size gives us a straightforward, ecologically relevant way to manipulate stop signalling.

We hypothesize that honeybee colonies of different sizes are differentially reactive to stop signals because the level of signalling to which each individual bee is exposed—and thus how sensitive each bee is to stop signals—depends on colony size. To test this hypothesis, we measured the effect of artificially generated stop signals on the level of waggle dancing in honeybee colonies of varying sizes. We predicted that waggle dancers in smaller colonies would be less responsive and should, therefore, exhibit less waggle dancing inhibition when compared with waggle dancers from larger colonies, when exposed to the same level of artificial stop signalling.

## 2. Material and methods

### 2.1. Colony size and responsiveness to artificial signals

**Figure 1.**
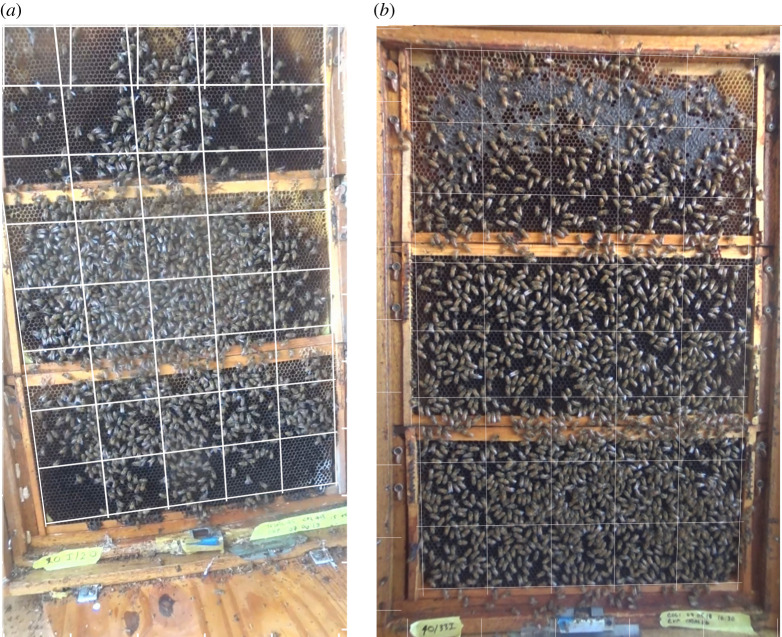
(*a*) Small colony with clustering visible (*n* = 984). (*b*) Large colony with more uniform comb coverage (*n* = 1400).

We used six colonies of *Apis mellifera ligustica* (four in 2018 and two in 2019), housed in three-frame wood observation hives (figure 1) with access to the outside through 4 cm diameter tubes inside a room at the University of California San Diego apiary. The observation colonies consisted of three standard wooden frames from Langstroth hives stacked on the top of each other and placed into a larger rectangular frame, to create one large rectangular surface. We maintained three to four colonies at any given time in this manner. Except during experimental trials, the two large surfaces were enclosed by wooden frames covered in plastic (windows), on the top of which was a layer of styrofoam insulation. Each plastic window and insulation were kept in place by removable wooden doors on each side of the colony (figure 1). A hole was cut at the bottom of the large frame and connected to a tunnel leading to the outside from the bottom of the table (the ‘entrance’), and each colony had its own entrance. The entrance was only accessible from one side of the colony, which we call the ‘front’ side of the colony.

The hives were constructed with a gate that directed traffic to one side of the hive. Bees could pass from one side to another only by walking to a gap at the top of the uppermost comb, a situation which led to the formation of the dance floor on the most accessible hive side and also concentrated stop signals on this side [10].

All colonies were in good health as determined by standard visual inspections [25]. Bees were allowed to enter and exit through tubes piercing the walls. Data were collected from June to August during 2018 and 2019. Given the highly disruptive nature of artificially increasing or decreasing colony size, we allowed the colonies to vary in size naturally. This meant that most colonies fell into both the ‘large’ and ‘small’ categories at various points (table 1). This naturalistic approach has drawbacks in terms of control, since we do not know the precise reasons that colonies increased or decreased in size and, therefore, differences we found might be because of some factor that is confounded with signalling or colony size. We felt, however, that artificially manipulating size was an even less favourable option, since we ultimately wish to draw conclusions about the mechanisms operating in networks that have formed organically.

**Table 1.** Colonies with observations in each treatment combination. Note that the data from 1B and 4B were collected in 2019, whereas all other data were collected in 2018. Sample sizes refer to the total combined number of samples in each group.

	control (no playback)	experimental (playback)
small	1B, 2, 3, 4, 4B *n* = 12	1, 2, 3, 4, 4B *n* = 13
large	1, 1B, 2, 4 *n* = 13	1, 1B, 2, 4, 4B *n* = 15

### 2.2. Artificial stop signal

The construction and testing of the artificial stop signal playback apparatus is published in [26]. Briefly, *A. mellifera* stop signals have a peak-to-peak vibrational displacement of 1.5 mm at 320 Hz [27]. Artificial vibrational signals, 200 ms pulses of a pure 340 Hz tone (within the range of variation of natural *A. mellifera* stop signals) were delivered to the comb directly adjacent to every waggle dancer in a colony using a small vibrational exciter (speaker with vibrating plastic tip mounted on the cone) attached to a light wood wand. This probe produced no detectable particle velocity sound at the probe tip located 25 mm from the speaker surface (details in [26]). During signal delivery, the tip was pressed into the comb, which caused the substrate in the immediate vicinity to vibrate. These were calibrated with a laser Doppler vibrometer (Polytec OFV3000 controller unit with an OFV502 laser head) to ensure that bees standing within one cell diameter of the probe would experience a displacement of 1.5 mm peak-to-peak.

Although stop signallers typically deliver their signals directly to another bee, they can also deliver signals to the comb [12,26]. As with *A. cerana* stop signal playback experiments [26], we found that stop signals reliably elicited a stereotypical freezing response when delivered next to a bee. However, direct contact of the probe to the bee usually elicited an escape response. This is likely due to the force exerted by the probe, which the experimenter rapidly moves to track dancing bees and can easily exceed natural stop signal forces. These problems are reduced when the vibrations are delivered to the comb since the targeted bee only senses the signals transmitted via the comb.

The natural rate of stop signals per dance circuit is approximately 0.16 stop signals/dance circuit (range of 0–2 stop signals/dance circuit). The estimate was computed from data collected using observation colonies at the University of California San Diego apiary from 2013 to 2014. The full dataset has been included with this paper. Specifically, we counted the number of waggle circuits in 213 waggle dances performed by naturally foraging bees from dance start to finish (beginning after a bee entered the nest from a foraging bout and ending when she stopped waggle dancing or left the nest for another foraging trip). The number and timing of stop signals was also noted. Because no manipulations were performed on the dancers, our estimate likely represents the overall rate of stop signals across a range of foraging conditions. Because we wanted to signal strongly enough to observe an effect, we aimed for a rate of roughly 0.5 stop signals/dance circuit, a higher rate that is, nonetheless, within the natural range.

### 2.3. Colony size

In addition to the waggle dance information, videos were also used to estimate colony size by extracting a still video frame from the 10 min pre-test baseline portion of each trial, correcting the fisheye lens distortion and then superimposing a 5 × 8 grid of 40 squares over the entire three frames of the observation colony using Gimp image editing software (v. 2.10) (figure 1). We then counted all bees visible on the surface of the colony. When multiple squares on the grid were completely full, we counted the bees within one full square, and multiplied by the number of squares. If the squares were not completely full, we counted all the bees in that square.

**Figure 3.**
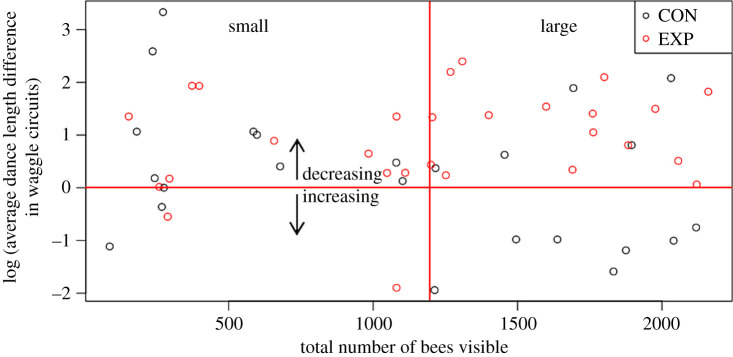
Log of the average dance length differences, measured in waggle circuits, between the pre-test baseline and the playback phases. Negative values on the *y*-axis indicated that the dances got longer, whereas positive values indicate the dances got shorter. The vertical red line represents 1200 visible bees, the upper limit for inclusion in the ‘small’ colony category.

Our observation colonies ranged in size from 88 to 2160 bees visible across the three frames. The surface of our largest colonies were completely covered in tightly packed bees, whereas our smallest colonies had a single small cluster, and, although this does not capture the full range in terms of possible sizes of colonies, it does represent about the maximum variability possible in a three-frame observation hive. Because we had no *a priori* cut-off for distinguishing between large and small colonies, we used three criteria. First, we used relative subjective dispersal of bees on the surface of the comb. Although the distinction was qualitative, we determined that at approximately 1200 bees, there was an observed shift from bees grouped in clusters to a more uniform distribution of bees on the comb (figure 1). Second, our 1200 cut-off also meant that about half of the observations fell into each size category, so the data in each category were balanced (figure 3 and table 1). Finally, we ran our analyses using cut-offs of 1000 and at 1500, but we failed to see effects using these parameters (see electronic supplementary material, S.6).

We considered the possibility that time was confounded with season in our design. This would be the case if, for example, all of the colonies grew over the two months during which they were filmed each year. However, among our six colonies, we observed: (i) one colony that remained in the large category, except for one trial, during which it was considered small, (ii) two colonies that started off large and then became small, with about half of the observations coming from each category, (iii) two colonies that remained mostly small, except for a few trials during which they were considered large, and (iv) two colonies that started out small, became large, and then returned to being small again.

**Figure 2.**
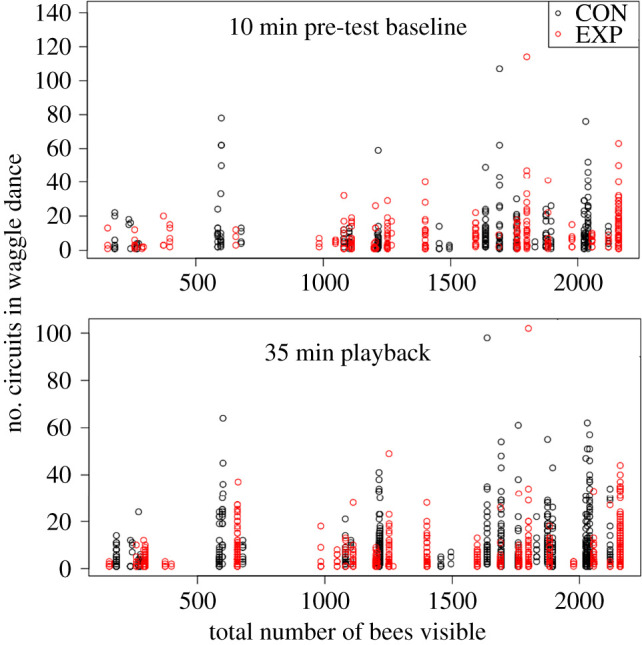
All recorded waggle dances, expressed as the number of dance circuits, during the 10 min baseline pre-test (top; control *n* = 266, experimental *n* = 298) and the 35 min playback (bottom; control *n* = 676, experimental *n* = 859). Dances that occurred during control trials are black and those that occurred during experimental trials are red.

Our full dataset included 62 total trials. However, in our analyses, we included only 53 trials (2099 total waggle dances), because we did not include trials in which there was no dancing in the pre-test phase (figure 2). Although seven of the nine excluded trials were small colonies, our final sample contained roughly equal numbers of observations from small and large colonies (table 1). Table 2 details the number of dances per group for Model 1 of our statistical analysis.

**Table 2.** Number of waggle dances observed by group.

treatment	colony size	time of day	waggle dances
control	large	afternoon	444
control	large	morning	254
control	small	afternoon	167
control	small	morning	77
experimental	large	afternoon	335
experimental	large	morning	513
experimental	small	afternoon	97
experimental	small	morning	212

### 2.4. Statistical analyses

All statistical analyses were conducted in R (v. 3.6.3) [28] using R studio (v. 1.3.595). We assessed the levels of artificial stop signals to the colonies of different sizes, and the rate of dancing across the different colony sizes using *t-*tests.

#### 2.4.1. Mixed models

*Model 1.* The first model included the raw dance lengths (measured as the number of waggle circuits) as the response; colony and trial as random effects; and treatment, colony size, test phase (pre-test baseline or playback) and time of day as categorical fixed effects. Model 1 was a generalized linear mixed model, specified with *glmer()* using the Nelder–Mead algorithm, with a Poisson identity function and a Wald test for assessment of the model effects. Generalized linear mixed models handle unbalanced designs well, and the Poisson identity function is appropriate for the analysis of count data.

*Model 2.* In order to better understand our first model, we collapsed our response variable across each trial, such that our outcome measure was the difference in the average dance length (measured in circuits) between the pre-test baseline and playback test phases for each trial. A negative number indicated that the average dance length increased, whereas a positive number indicated a decrease in the average dance length. We did not include any trials for which there was no dancing during the 10 min pre-test baseline period. This left us with 26 observations in the control condition (no artificial signals delivered), and 28 observations in the experimental condition (artificial stop signals delivered, table 1). A qq-plot and accompanying Shapiro–Wilk test (*shapiro.test()*) indicated that the error of the average difference in dance length was not normally distributed (*W* = 0.864, *p* < 0.0001), so we log-transformed the absolute values of the average dance length difference and re-applied the original sign after transformation to preserve the direction of the difference. A Shapiro–Wilk test on the transformed data indicated that the error was not significantly different from normal (*W* = 0.99, *p* = 0.75). We fit a model with log-transformed dance length differences as the response variable; colony as a random effect; and treatment, colony size and time of day as categorical fixed effects. Model 2 was specified with *lmer()*, and the model effects were tested using Satterthwaite's method to generate error terms for *F*-statistics [29] with Type III sums of squares.

## 3. Results

Means are reported as ±1 s.d. We verified that we had delivered equal rates of artificial stop signals per dance circuit to large and small colonies, which were 0.40 ± 0.24 signals per dance circuit for large colonies and 0.48 ± 0.27 for small colonies (Welch's *t*_23.97_ = 0.83, *p* = 0.42). During the stop signal playbacks, the mean number of waggle dances observed in large colonies, corrected for colony size, was 0.023 ± 0.02 dances per bee, and 0.037 ± 0.05 dances per bee in small colonies, a non-significant difference (Welch's *t*_15.85_ = −0.92, *p* = 0.37).

### 3.1. Model 1

Using Wald *χ*^2^ tests, we detected a number of statistically significant model predictors and interactions in Model 1, and these are summarized in table 3.

**Table 3.** Summary of Model 1 significant fixed effects based on Wald *χ*^2^ (*α* = 0.05).

fixed effect	estimate	s.e.	*Z*-value	*p*-value
colony size	−0.11	0.04	−2.77	0.006
treatment	−0.60	0.04	−16.83	<0.0001
colony size × time of day	−0.38	0.10	−3.64	0.0003
treatment × time of day	0.17	0.05	3.50	0.0005
colony size × trial phase	0.29	0.06	4.90	<0.0001
time of day × trial phase	0.22	0.05	4.099	<0.0001
colony size × treatment × time of day	0.46	0.16	2.86	0.004
colony size × treatment × trial phase	−0.36	0.13	−2.73	0.006
treatment × time of day × trial phase	−0.36	0.08	−4.70	<0.0001

The significant interaction between Colony size, Treatment and Trial phase (*Z* = −2.73, *p* = 0.006) supports our hypothesis. However, given the large number of other significant effects, interpretation of these results is not straightforward. Therefore, for our next analysis, we simplified the model by collapsing the data into a single difference score for dance length per trial between the pre-treatment baseline trial phase and the stop signal playback phase.

### 3.2. Model 2

**Figure 4.**
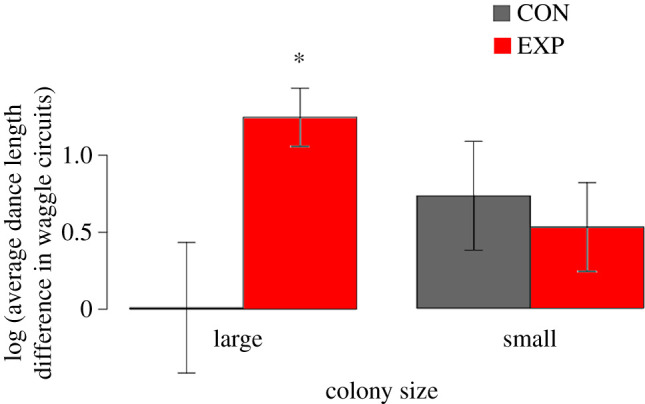
Means and 95% confidence intervals of the log-transformed difference scores for large and small colonies across the control (no playback) and experimental (artificial stop signal playbacks) treatment groups, *F*_1,38.53_ = 4.48, *p* = 0.04, based on Saitherwaite's method and Type III sums of squares. Positive values indicate increased inhibition (shorter dance length) during the playback phase of the experiment.

In order to satisfy the normality assumption, we log-transformed our average dance difference scores and re-applied the original sign to the transformed scores (figure 3). We used Satterthwaite's method to generate *F*-statistics to test the Model 2 effects. We detected a significant interaction between Treatment and Colony size (*F*_1,38.53_ = 4.48, *p* = 0.04, figure 4 and table 4), supporting our prediction that smaller colonies would exhibit less waggle dance inhibition in the presence of stop signals than large colonies. We did not detect any other significant model effects (table 4).

**Table 4.** Tests of Model 2 effects, based on *F*-statistics generated using Saitherthwaite's method and Type III sums of squares.

fixed effect	mean square	numerator d.f.	denominator d.f.	*F*-statistic	*p*-value
treatment	2.85	1	38.68	2.26	0.14
colony size	0.40	1	17.24	0.32	0.58
time of day	0.40	1	41.15	0.32	0.57
treatment × colony size	5.65	1	38.53	4.48	0.04^a^
treatment × time of day	4.54	1	44.99	3.60	0.06
colony size × time of day	0.16	1	39.30	0.13	0.72
treatment × colony size × time of day	0.25	1	45.00	0.20	0.66

^a^Statistically significant at *α* = 0.05*.*

## 4. Discussion and conclusion

Our finding that large colonies were more sensitive to the inhibition provided by stop signalling, when compared with small colonies, is somewhat counterintuitive. However, the specific threat posed by predators or a deteriorating food source to a honeybee colony depends on a number of factors, including how large and well established the colony is. For example, a larger colony might only be marginally affected by curtailing foraging at a dangerous or crowded site that is otherwise profitable, because it has sufficient foragers to cover multiple sites, and enough food stores to buffer against variable rates of resource intake. By contrast, ceasing foraging at a dangerous or deteriorating site might be more costly for a small colony with few foragers available to locate and exploit alternative food sources and less food stores. Although we did not quantify food stores in our colonies and cannot completely rule out this possibility, it is unlikely that the observed changes in waggle dance inhibition were directly driven by the level of stored pollen or nectar. All of our colonies were three-frame observation colonies with little space in which to accumulate food stores. Of the photos we took in which the comb is not totally obscured by bees, no colonies had amassed more than 1/3 frame of pollen or capped honey.

In nature, honeybee colonies reproduce by colony fission, with a fraction of the colony, the swarm, leaving to found a new colony [30]. Colonies thus begin small with essentially no food stores—a relatively perilous state of affairs because small disturbances such as being unable to locate a profitable foraging site on any given day can have major consequences. Small colonies must expand to the point that enough workers are available for all basic tasks (e.g. brood-rearing) before foragers are able to generate the food surpluses that buffer against variable rates of resource intake. In fact, research and modelling demonstrates that colonies need a minimum size to survive [30]. Our finding that weak or small colonies should resist switching away from foraging at dangerous or crowded locations more than strong colonies aligns with the information primacy hypothesis [31], and hungry bees have been shown to favour exploitation over exploration [32].

To protect against the outsized influence of perturbations, individuals in small colonies could become less responsive to signals that inhibit foraging, such as stop signals. For small colonies with little or no food stores, any inhibition on foraging might put the survival of the colony at risk, even if the site is suboptimal, and particularly if the site was initially sufficiently favourable to elicit waggle dancing. As the colony becomes larger and their food stores more established, becoming more sensitive to stop signals may allow the colony to optimize its foraging to take advantage of only the most profitable locations.

### 4.1. A widespread phenomenon?

All biological systems are capable of *adaptation*, the capacity to respond to changing conditions. Although much remains unknown, network-level adaptation has been most thoroughly investigated in nervous systems, in which adaptation arises, in part, from plasticity in neural synaptic connections. Multiple synapse types have properties that change in response to increased or decreased frequency of signal transmission between neurons across various timescales, and these changes can result in either increased or decreased synaptic efficacy [33].

Recent work in statistical mechanics suggests that large, sparsely coupled artificial networks are more robust (less reactive) than small, sparsely coupled networks [34]. However, there is at least one key difference between these types of networks and biological networks: plasticity in the connections between the nodes. Some neuroscientists have argued that synaptic plasticity, because it represents change, is necessarily distinct from and antithetical to nervous system stability [35]. However, others have recognized the role of plasticity as a homeostatic mechanism for maintaining network-level stability [36,37]. For example, acquired drug tolerance has been hypothesized to result from nervous system plasticity meant to maintain consistent levels of neurotransmitter system activity but now inappropriately influenced by exogenous ligands [38].

Although others have described context-dependent plasticity in biological networks, such as socially mediated behavioural plasticity in groups [39–41], to the best of our knowledge, no one has previously considered that network size might drive plasticity in the network elements themselves. Intriguingly, larger bumblebee colonies have been observed to respond more quickly to perturbations of in-hive carbon dioxide levels than smaller colonies, despite a similar proportion of the workforce being dedicated to the effort across all colonies, but no specific mechanism for this difference was suggested [42]. A re-reading of existing neuroscience literature also provides hints of network size-dependent plasticity in other systems.

### 4.2. Evidence for size-dependent plasticity in biological neural networks

In both honeybee colonies and nervous systems, robust and adaptive information processing is carried out by distributed networks of heterogeneous components exchanging excitatory and inhibitory signals [43]. Both also undergo significant changes of size with respect to the number of elements over time. In neuronal networks, these changes occur on multiple timescales, and depend on modifications of the structural and functional connectivity among the constituting neurons [44]. Moreover, changes of neuronal circuit size and neuronal responsiveness are co-regulated, and are related to the robustness of neuronal network activity to external perturbations.

For example, small and immature networks exhibit a stereotypical pattern of oscillatory activity, known to be important for promoting axonal growth and synapse formation during development. The individual neurons in these networks exhibit low responsiveness and make these oscillatory patterns robust to perturbations [45–47]. As these circuits grow in size and connectivity, the responsiveness of their constituting neurons increases, resulting in more variable and complex spatio-temporal patterns of network activity observed *in vitro* [48] and *in vivo* [49]. Similar co-regulation of network size and neuron responsiveness is observed during sleep and anaesthesia, during which networks become functionally de-coupled [50–52], and in the development of Parkinson's disease, which is characterized by the reduction in the size of networks in some brain regions [53–55]. Thus, our data showing that bees in smaller colonies are less responsive to signals appear to mirror what occurs in small networks of neurons.

### 4.3. Implications for artificial network design

Although plasticity is ubiquitous in biological networks, it is conspicuously absent in artificial ones (e.g. computer networks). In these systems, distributed robustness is usually achieved by increasing network size in order to introduce redundancy and degeneracy [56]. We propose that, if size-dependent plasticity is related to maintaining network stability, including size-dependent tuning parameters for connection strength might offer a more efficient solution to the problem of small network instability, and one that is flexible in the face of network expansion.

The empirical evidence presented in this paper suggests that honeybee colonies exhibit size-dependent behavioural plasticity with respect to their individual responsiveness to stop signals. Although our findings make sense in the light of the natural ecology of large and small honeybee colonies, we have yet to directly test the functional consequences of this behavioural plasticity on foraging. Additionally, we have cited examples from neuronal networks that suggest size-dependent plasticity with respect to the sensitivity to signals might be a widespread phenomenon in biological systems because it maintains network stability. However, this hypothesis needs to be rigorously tested.

## 5. Limitations of the study

Communication is inherently noisy, and thus efforts to detect patterns that arise from small effects are hampered by small samples. Stop signals are only weakly inhibitory, and should, therefore, produce relatively small effects on the overall behaviour of the colony. Thus, a limitation of our study is the small sample size due to the large amount of time and effort required to collect such data. In our playback experiments, individual dancers need to be manually tracked, and the artificial stop signal must be precisely delivered next to the rapidly moving dancer. In our censuses of waggle dancing, we needed to manually score sound and video data because of limitations and inaccuracies in automated video detection. Recent advances have been made in automated detection algorithms [60]. However, the current tools still have substantial limitations, including the inability to accurately decode shorter dances, and the need to manually track bees to avoid high error rates. Once it is perfected, automated software should significantly increase the ability to test hypotheses using full honeybee colonies.

A second limitation is that although we have shown an effect of colony size, this study does not directly test the functional consequences of such a difference. Many possibilities exist for how manipulating signalling might affect colony-level foraging behaviour. Colonies are systems of interacting individuals that adapt to changing conditions to maintain levels of biologically relevant variables, such as internal food stores [61]. It is difficult to precisely predict the effects of altering one aspect of an interconnected network, since altering one aspect necessarily affects other aspects. There is no reason to suspect *a priori* that such a manipulation will map in a straightforward way onto the output of the system (e.g. a linear effect). Although it is perhaps more apparent in honeybee colonies, where the relationships between the elements are more immediately visible, this is a general limitation of any study involving a living system [62,63].

## Data accessibility

The three datasets discussed in this paper are accessible here: https://doi.org/10.5281/zenodo.5474927. The R script used for the statistical models is included in the electronic supplementary material.

The data are provided in electronic supplementary material [64].
